# New type of interaction between the SARAH domain of the tumour suppressor RASSF1A and its mitotic kinase Aurora A

**DOI:** 10.1038/s41598-019-41972-x

**Published:** 2019-04-03

**Authors:** T. Szimler, É. Gráczer, D. Györffy, B. Végh, A. Szilágyi, I. Hajdú, P. Závodszky, M. Vas

**Affiliations:** 1 Institute of Enzymology, Research Centre for Natural Sciences, Hungarian Academy of Sciences, Magyar tudósok krt. 2., H-1117 Budapest, Hungary; 2 ELTE NAP Neuroimmunology Research Group, Department of Biochemistry, Institute of Biology, Eötvös Loránd University, Pázmány Péter sétány 1/C, H-1117 Budapest, Hungary

## Abstract

The tumour suppressor protein RASSF1A is phosphorylated by Aurora A kinase, thereby impairing its tumour suppressor function. Consequently, inhibiting the interaction between Aurora A and RASSF1A may be used for anti-tumour therapy. We used recombinant variants of RASSF1A to map the sites of interaction with Aurora A. The phosphorylation kinetics of three truncated RASSF1A variants has been analysed. Compared to the RASSF1A form lacking the 120 residue long N-terminal part, the *K*_m_ value of the phosphorylation is increased from 10 to 45 μM upon additional deletion of the C-terminal SARAH domain. On the other hand, deletion of the flexible loop (Δ177–197) that precedes the phosphorylation site/s (T202/S203) results in a reduction of the *k*_cat_ value from about 40 to 7 min^−1^. Direct physical interaction between the isolated SARAH domain and Aurora A was revealed by SPR. These data demonstrate that the SARAH domain of RASSF1A is involved in the binding to Aurora A kinase. Structural modelling confirms that a novel complex is feasible between the SARAH domain and the kinase domain of Aurora A. In addition, a regulatory role of the loop in the catalytic phosphorylation reaction has been demonstrated both experimentally and by structural modelling.

## Introduction

The tumour suppressor gene Ras-association domain family 1 isoform A (RASSF1A) is frequently silenced in a wide range of cancers. The exact mechanism by which RASSF1A exerts its tumour suppressor effects has not been clarified^1–3^. RASSF1A protein is involved in three important cellular processes: microtubule stability, mitosis and induction of apoptosis. Loss of function of RASSF1A leads to accelerated cell cycle progression and resistance to apoptopic signals, resulting in increased cell proliferation. Thus, the development of targeted drugs to restore RASSF1A function is a desirable goal. In order to achieve this, the phosphorylation status of RASSF1A, sensitively influencing its various functions should be controlled.

RASSF1A is known to be phosphorylated by certain cellular kinases, such as the mitotic kinase Aurora A^4,5^ or protein kinase A^6^. Phosphorylation of RASSF1A by Aurora A on Thr 202 and/or Ser 203 disrupts its association with the microtubule network, thereby allowing mitotic progression^4^. The Aurora A mediated phosphorylation also suspends the mitotic blockade caused by RASSF1A through interaction with the complex of Anaphase-Promoting Complex and Cell division cycle protein 20 (APC/CCdc20)^5^. Furthermore, the activated association of APC/CCdc20 ubiquitylates RASSF1A, priming it for degradation. Thus, Aurora A mediated phosphorylation of RASSF1A promotes mitotic progression by causing APC/CCdc20 activation and subsequent degradation of RASSF1A^7^.

The mitotic Aurora A kinase is often targeted by specific inhibitors as potential drugs against cancer^8–11^. However, inhibiting a multifunctional protein might cause disruption of its other essential physiological functions. For this reason, targeting of a particular protein-protein interaction, such as Aurora A kinase-RASSF1A enzyme-substrate interaction could be the solution. Although there are numerous structural and functional studies with Aurora A kinase^12–15^, information on the isolated RASSF1A and its functional complex with Aurora A is still lacking. Up to now, interaction of RASSF1A with Aurora A kinase has only been demonstrated qualitatively by pull-down experiments^4,16^.

**Figure 1:**
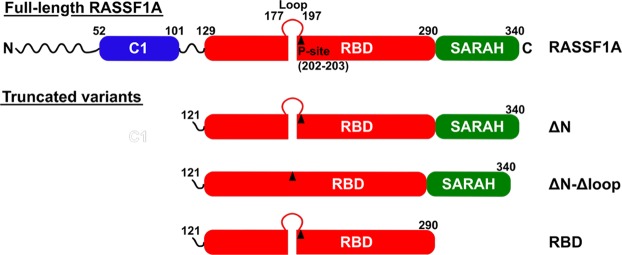
Structural scheme of RASSF1A and its truncated variants. The topology of RASSF1A domains and other important molecular regions are presented schematically. Domains are represented by rounded rectangles, while unstructured regions are shown as curved lines. A black triangle marks the position of the Aurora A phosphorylation site. Numbers represent positions of residues bordering each structural region. RASSF1A has an N-terminal unstructured region with a small C1 domain. This region is missing from each truncated mutant. The largest domain of RASSF1A is the Ras binding domain (RBD), which includes the Aurora A phosphorylation site (P-site) preceded directly by a flexible loop. The C-terminal part of the molecule consists of an unstable α-helical SARAH domain.

In this work, we aim to quantitatively characterise the enzyme-substrate interaction between the isolated Aurora A kinase domain and RASSF1A using an *in vitro* kinetic phosphorylation assay. We found that the isolated full-length RASSF1A exhibited an extremely strong tendency for aggregation. This may be due to the long unstructured N-terminal region (Fig. 1) which otherwise may be involved in fuzzy protein-protein interactions^17,18^. Because our study required well-defined soluble proteins, we have expressed a truncated mutant of RASSF1A by deleting the N-terminal 120 residues, yielding the fragment ΔN (Fig. 1). Although this protein fragment is identical to the C-terminal part of RASSF1C, another important member of the RASSF1 family of proteins^3,19^, there are also crucial differences between RASSF1A and RASSF1C which prohibit generalisation of our results to both these proteins. First, while the *in vivo* phosphorylation of RASSF1A by Aurora A kinase is well documented^5^, no similar findings have been reported for RASSF1C. Second, there is accumulating evidence in favour of the oncogenic character of RASSF1C, in contrast to the tumour suppressor effect of RASSF1A^20^. Correspondingly, the *in vivo* spatial-temporal localisations of the two RASSF1 proteins (A and C) are probably entirely different.

The ΔN fragment consists of the Ras-binding domain (RBD), where the phosphorylation site is located, and the attached C-terminal Sav/Rassf/Hpo (SARAH) domain, a domain often involved in protein-protein interactions^2^. Additional deletion of the SARAH domain yielded RBD, which was also examined in order to test the contribution of SARAH domain to the catalytic complex formation with Aurora A. Another aspect of our kinetic studies was to test the previously assumed role^21^ of the flexible loop (residues 177–197) preceding the phosphorylation site/s (T202/S203) in the functional complex formation with Aurora A. For this purpose, the loop-deleted variant of ΔN (i.e. ΔN-Δloop) was also expressed and studied. All these truncated variants are schematically illustrated in Fig. 1. Based on detailed kinetic analysis of phosphorylation of these RASSF1A constructs by Aurora A and on structural modelling, we make an attempt to localize critical protein-protein interaction site(s) and confirm this by more specific binding studies. Such sites may facilitate the design of small molecular weight specific inhibitors to prevent unwanted phosphorylation of RASSF1A without inhibiting the kinase activity of Aurora A.

## Results

### The dimerization state of the different RASSF1A variants

**Figure 2:**
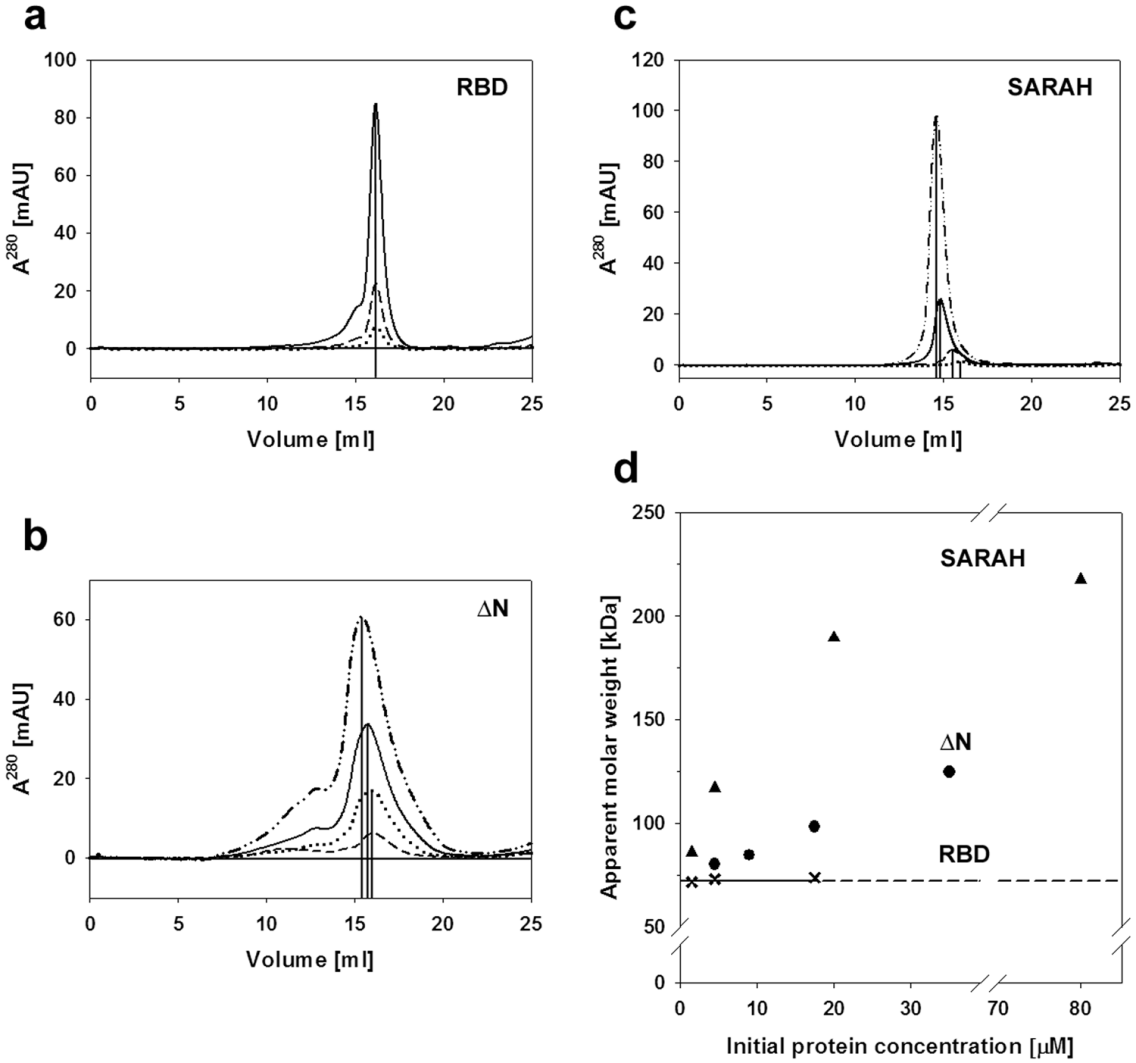
Assessing the oligomerization state of the ∆N, RBD and SARAH constructs by size exclusion chromatography. The constructs (all fused to an MBP tag) were diluted to different concentrations. 100 μl volumes of each sample were injected to a Superose 6 10/300 GL column. Initial concentrations of the samples: (**a**) RBD: 17.5 μM (solid line), 4.5 μM (dashed line) and 1.5 μM (dotted line). (**b**) ∆N: 35 μM (dash-dot line), 17.5 μM (solid line), 9 μM (dotted line) and 4.5 μM (dashed line). (**c**) SARAH domain: 80 μM (dash-dot line), 20 μM (solid line), 4.5 μM (dashed line) and 1.5 μM (dotted line). All proteins eluted in one major peak. In the case of RBD and ∆N variants some inhomogeneities appear at higher molecular masses. The elution volumes for these main peaks are marked by vertical lines. These were the same for all RBD samples, but were dependent on concentration in the case of ∆N and the SARAH domain. (**d**) Apparent molecular weights corresponding to each peak (determined using appropriate calibration data) are plotted against the initial concentrations and also presented in Table 1. The apparent *M*_w_ of RBD (✖) is very similar to that of the monomeric protein (63 kDa) and independent of its concentration. In contrast, the apparent *M*_w_ of ∆N (●) and SARAH (▲) are both shifted from the *M*_w_ of the monomer (69 and 51 kDa, respectively) towards higher molar weigths with the increasing concentrations. These data show that the ∆N exists as an equilibrium mixture of its monomeric and dimeric forms, and this dimerization occurs via the SARAH domain. This equilibrium should be relatively quick, since the two states cannot be separated using gel filtration. It is also worth noting that dimers of both ∆N and the SARAH domain elute at lower volumes than those expected based on their molar weights. This is likely due to the elongated shape of the homodimers.

It is known that SARAH domain containing proteins can form homo- and heterodimers through this domain^22,23^. To determine if the RASSF1A variants used in this study form dimers, we performed analytical size-exclusion chromatographic experiments. Since the dimerization state of proteins is dependent of their concentration, the ∆N and RBD constructs were diluted to a series of different concentrations. These samples were then injected to a Superose 6 column. The resulting chromatograms are shown in Fig. 2a,b. As a control experiment, we also carried out chromatography with the separately expressed SARAH domain of RASSF1A (Fig. 2c).

The protein samples of ∆N and RBD are eluted in a single major peak, with some inhomogeneities detectable at higher molecular masses, indicating their propensities for easy aggregation. The SARAH domain construct was also eluted as a single peak. The chromatographic column was calibrated using a series of proteins of known molar mass. The calibration data was then used to determine the molecular masses corresponding to each peak (Table 1). In the case of RBD, all three samples with different concentrations eluted at the same volume (Fig. 2a, marked by the vertical line), closely corresponding to the molecular mass of the MBP-tagged RBD (i.e. 63 kDa). However, the elution volume for the constructs of ∆N and the SARAH domain turned out to be dependent of the initial sample concentration, with the higher concentration samples eluted at lower volumes (Fig. 2b,c) corresponding to higher average molecular masses, as illustrated in Fig. 2d.

**Table 1:** Concentration dependence of apparent molecular weight of the various RASSF1A mutants, as determined by size-exclusion gel-chromatography.

RASSF1A	Proper *M*_w_	Apparent *M*_w_ (kDa) at initial concentrations of:						
mutant	(kDa)	1.5 μM	4.5 μM	9.0 μM	17.5 μM	20 μM	35 μM	80 μM
RBD	69.3	71.6	73.1	—	73.7	—	—	—
∆N	63.2	—	80.3	84.5	98.3	—	124.8	—
SARAH	50.1	85.7	116.9	—	—	189.6	—	217.5

Thus, the size exclusion chromatographic experiment with RBD clearly indicates the existence of its monomeric form, at least within the investigated protein concentration range. For the ∆N variant, as well as for the isolated SARAH domain, the experiments indicate equilibrium between monomeric and dimeric states, with association/dissociation rates faster than the rate of their possible chromatographic separation. It is evident that the dimerization ability of ∆N is due to its SARAH domain.

### The effect of RASSF1A deletions on the kinetics of phosphorylation by Aurora A

To study the functional interaction between Aurora A and RASSF1A, *in vitro* phosphorylation experiments were performed on deletion mutants of RASSF1A. A constant concentration of Aurora A was incubated with various concentrations (up to 20 μM) of each RASSF1A mutant in the presence of MgATP partially labelled with radioactive ^32^P on its γ-phosphate group. By measuring the intensity of radioactive labelling incorporated into the RASSF1A substrate, the amount of phosphorylated RASSF1A could be calculated.

**Figure 3:**
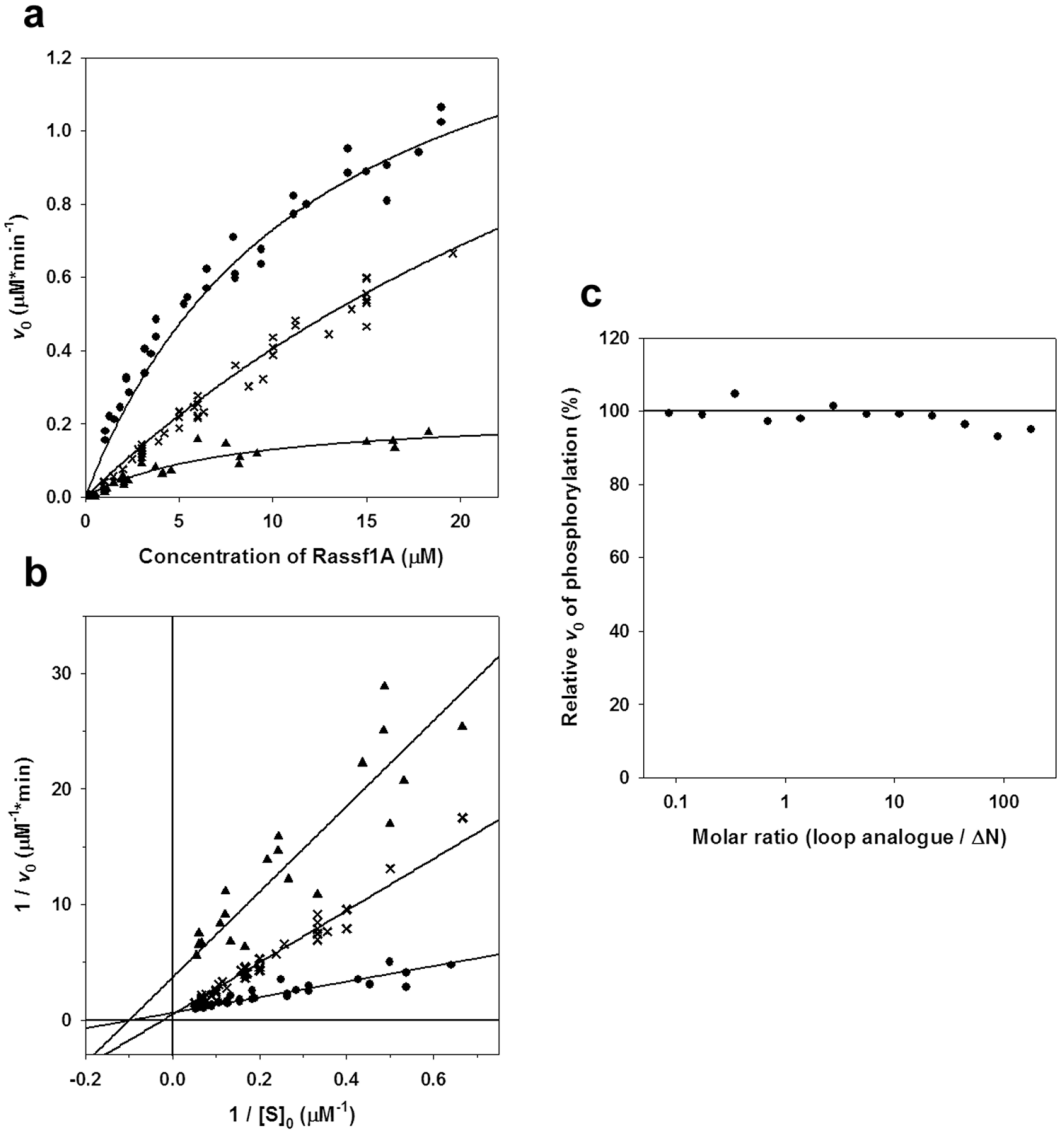
Phosphorylation kinetics of RASSF1A by Aurora A. The initial velocities of phosphorylation of the RASSF1A ∆N (●), ∆N-∆loop (▲) and RBD (✖) are plotted against their concentrations either as a Michaelis-Menten hyperbolic (**a**) or as a linearized Lineweaver-Burk plot (**b**). The concentration of Aurora A kinase domain was kept at a constant 40 nM in all experiments. All measurements were performed at a pH of 7.4, in the presence of 0.4 mM ATP (γ-^32^P labelled), 5 mM MgCl_2_, 100 mM NaCl and 2 mM DTT. The reaction mixtures were incubated for 2 minutes at 25 °C then stopped. The initial velocities for each reaction were calculated by detecting the intensity of radioactivity incorporation into the substrate RASSF1A. The fitted kinetic parameters are listed in Table 2. Data reveal that deletion of the loop (∆N-∆loop) significantly reduces the *k*_cat_ parameter of phosphorylation – compared to ∆N – without affecting *K*_m_. In contrast to this; *K*_m_ for the RBD variant is significantly higher than that of ∆N, while *k*_cat_ is also increased slightly. This can also be seen on the Lineweaver-Burk plot (**b**), where the x- and y-axis interrupts represent −1/*K*_m_ and 1/V_max_, respectively. These results show that the SARAH domain is important for the physical binding to Aurora A, while the loop is involved in the phosphorylation reaction. (**c**) Phosphorylation of the ∆N mutant in the presence of a synthetic peptide, identical to the loop. The concentration of ∆N was kept at a constant 5 μM, while that of the peptide was varied between 0.2–800 μM. The extent of phosphorylation is shown relative to that detected in a negative control experiment (with no peptide added), represented by a horizontal line (100%). Data are plotted against the molar ratio of the peptide to ∆N. No significant effect on the enzyme reaction by the peptide could be detected, showing that the loop in itself does not bind directly to Aurora A.

The data yielded by these experiments are illustrated on Fig. 3a as Michaelis-Menten hyperbolic plots. However, the corresponding kinetic parameters were determined from a Lineweaver-Burk plot of the data (Fig. 3b), since this method is less affected by the lack of measurements at higher substrate-concentrations. The kinetic parameters yielded by fitting these double-reciprocal plots are presented in Table 2.

**Table 2:** Kinetic parameters of phosphorylation of different RASSF1A variants by Aurora A.

RASSF1A mutant	*k*_cat_ [min^−1^]	*K*_m_ [μM]
∆N (below 20 μM)	35 ± 10	9.0 ± 2.5
∆N (above 20 μM)	70 ± 15	41 ± 5
RBD	47 ± 5	43 ± 5
∆N-∆loop	6.8 ± 0.6	10.0 ± 2

For RBD (which lacks the SARAH domain), the *K*_m_ value of the reaction is significantly increased compared to ∆N, suggesting that the SARAH domain increases the affinity of RASSF1A to Aurora A. This finding suggests direct physical binding of the SARAH domain to the surface of Aurora A. Deletion of SARAH domain also increases *k*_cat_ slightly. A possible explanation for this effect is that by stabilizing the E*S complex, the SARAH domain may also hinder its dissociation. This would also imply that – at least in the presence of the SARAH domain – dissociation may contribute to the rate limiting step of the reaction, i.e. the classical fast-equilibrium Michaelis-Menten mechanism may not entirely hold.

Comparison of the kinetic parameters determined for the ∆N and ∆N-∆loop variants shows no significant difference in *K*_m_, but *k*_cat_ is much smaller for ∆N-∆loop. These data suggest that – in contrast with the SARAH domain – the phosphorylation loop has an important catalytic role, probably by providing flexibility required for the formation of the catalytically competent E*S complex conformation. The unchanged *K*_m_ suggests that the loop itself does not contribute to the formation and stabilization of the physical interaction between RASSF1A and Aurora A.

To confirm this interpretation, we performed another phosphorylation experiment where Aurora A and RASSF1A ∆N were present in a constant concentration, and a synthetic peptide representing the loop (residues 177–197) was also added to the reaction mixture in varying amounts, up to a concentration of 1 mM. In this experiment, the presence of the peptide had no detectable effect on reaction velocity, proving its inability to disturb the formation of the E*S complex (Fig. 3c). Therefore, it can be safely assumed that the loop itself does not directly bind to Aurora A.

### The phosphorylation kinetics of the ∆N dimer is similar to that of RBD

**Figure 4:**
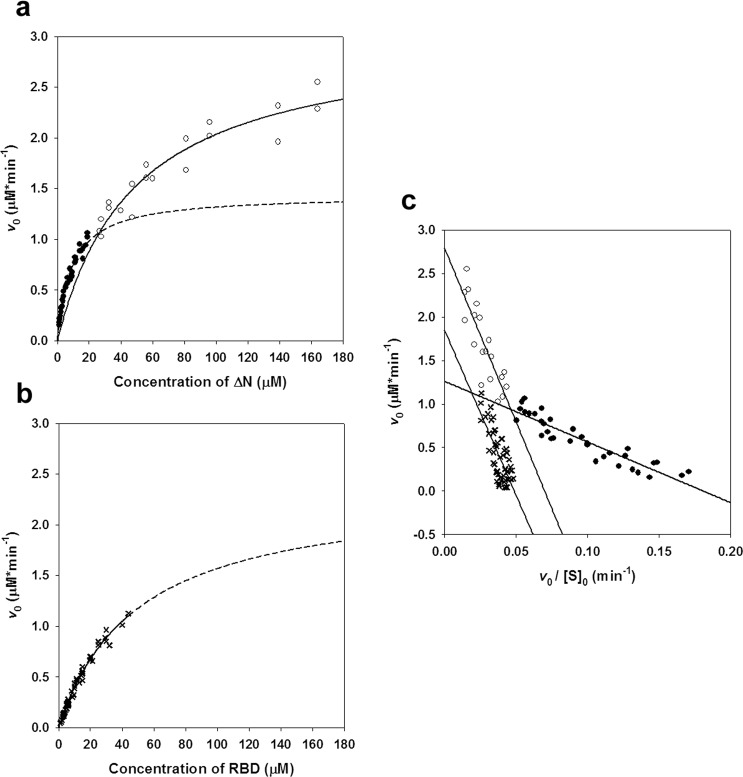
The monomeric and dimeric states of ∆N exhibit distinct kinetics. (**a**) The kinetic experiments shown in Fig. 3 were extended to higher concentrations (up to 160 μM) of RASSF1A ∆N. A curve fitted to the data of Fig. 3a (●, dashed line) strongly deviates from the actual measurements in the higher concentration range (○). Likewise, the Michaelis-Menten curve fitted to these higher-concentration data points (solid line) aligns poorly with the measurements in the lower-concentration range. This indicates that phosphorylation kinetics of ∆N cannot be described by a single Michaelis-Menten hyperbole. (**b**) Michaelis-Menten curve showing phosphorylation of the RBD construct in an extended concentration range. (**c**) Eadie-Hofstee plot of the kinetics data presented on subfigures (**a**,**b**). A single linear plot fits the RBD (✖) data well, showing that the phosphorylation of RBD exhibits Michaelis-Menten type kinetics. In contrast, ∆N (● and ○) phosphorylation kinetics is clearly biphasic, with an apparent breakpoint around 20–25 μM of ∆N concentration. At the higher concentration range, *K*_m_ (corresponding to the slope of the plots) is much greater, and basically identical to the *K*_m_ of RBD (Table 2). The dimeric form of ∆N – like RBD – lacks free SARAH domain, which explains their similar kinetic behaviours towards Aurora A.

The kinetic data presented in the previous section show that substrate saturation cannot be reached (especially for the RBD variant) using lower concentrations of RASSF1A. Also, it seemed desirable to investigate if dimerization of ∆N has any effect on phosphorylation kinetics. Therefore, the experiments were extended to a higher (possibly physiologically less relevant) concentration range in the case of the ∆N (up to 160 μM) and RBD (up to 45 μM) variants (Fig. 4). With ∆N, the Michaelis-Menten hyperbole fitted to measurements at lower concentrations (the same as on Fig. 3) showed a large deviation from the experimental data collected at higher concentrations (Fig. 4a). Similarly, fitting the data in the high concentration range resulted in a curve aligning very poorly to the lower concentration data points. The fact that kinetic data could not be fit by a single hyperbole in the whole concentration range suggested that phosphorylation of this mutant does not follow simple Michaelis-Menten kinetics. RBD, however, showed no such abnormal behaviour (Fig. 4b).

To further examine this effect, kinetic measurements were also visualized as an Eadie-Hofstee plot (Fig. 4c). In the case of RBD, the data can be fit by a single straight line, but for ∆N, the graph clearly reveals a biphasic character. The curve can be fitted quite precisely by a broken linear function, with a breakpoint between 20–25 μM substrate concentrations. Since RBD phosphorylation fits Michaelis-Menten kinetics well (at least in the studied concentration range), and it has been proven that this variant exists purely as a monomer, it is likely that the biphasic nature of ∆N phosphorylation kinetics is related to its dimerization state. If the monomer is dominant at lower concentrations, while the dimer is favoured at higher concentrations, it can be assumed that fitting the two linear stretches of the curve yields the (apparent) kinetic parameters for the monomer and dimer, respectively (Table 2).

Interestingly, the *K*_m_ for the dimeric ∆N is much higher than that determined for its monomeric state. This suggests that RASSF1A dimerization significantly hinders complex formation with Aurora A. Furthermore, the *K*_m_ value measured for RBD is remarkably similar to that measured for the ∆N dimer, indicating that dimer formation through the SARAH domain reduces binding affinity towards Aurora A to a similar extent as when SARAH is completely absent. This finding provides further evidence that the SARAH domain stabilizes the Aurora A – RASSF1A complex through a direct contact with Aurora A.

Compared to the monomer, the dimer also shows an increase in *k*_cat_, which can be explained by faster dissociation of the E*S complex in the absence of the SARAH-Aurora A interaction.

### Binding of the various RASSF1A forms to Aurora A

**Figure 5:**
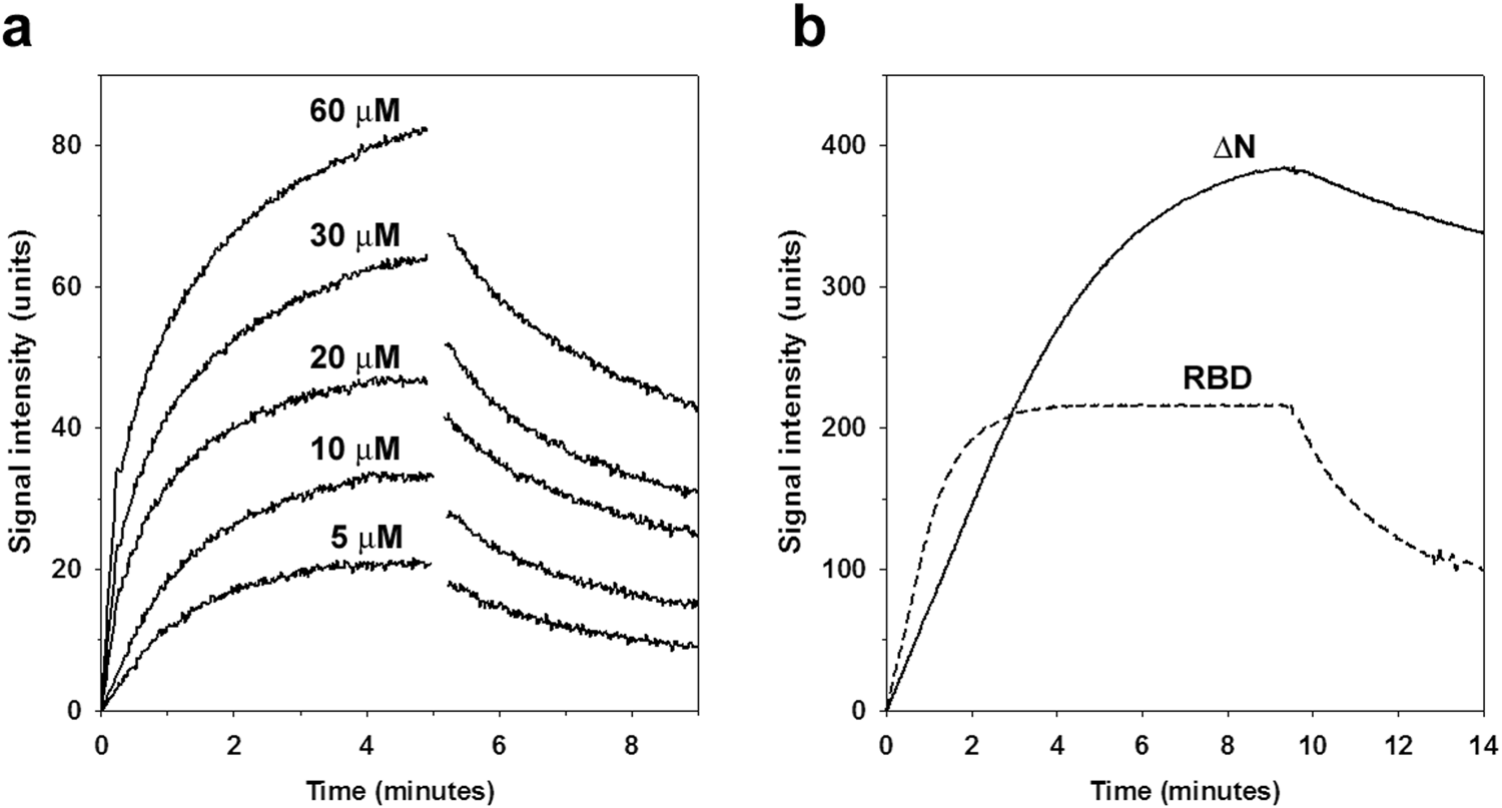
Binding between Aurora A and the RASSF1A mutants. SPR experiments were carried out to study the direct physical interactions of Aurora A with the different RASSF1A variants. Aurora A kinase domain was used as the immobilized ligand, while various concentrations of ∆N, RBD and the SARAH domain were injected as analytes. The resulting sensorgrams are shown as plots of the signal intensity (in arbitrary units) against time. The intensity values are corrected by the signal of a reference cell (with no ligand immobilized). (**a**) SARAH domain was injected at different concentrations indicated on the figures. The kinetic curves obtained show direct physical binding between the SARAH domain and Aurora A, which is dependent on the concentration of the analyte. (**b**) The binding kinetics of ∆N (solid line) and RBD (dashed line) are shown, both at an analyte concentration of 0.05 μM. RBD shows faster association to and also much faster dissociation from Aurora A than its ∆N counterpart. It is likely that the SARAH domain stabilizes the Aurora A – RASSF1A complex, resulting in slower dissociation, while it might also slightly hinder its formation. The higher signal maximum measured for ∆N shows that a larger amount of this variant was bound to the surface than RBD, probably due to the dimerization of ∆N, in contrast to RBD (cf. Fig. 2). As for SARAH domain, it binds to Aurora A in significantly smaller quantities than both ∆N and RBD, even at much higher concentrations. This shows that the affinity of SARAH towards Aurora A is much weaker than these other RASSF1A mutants, making it an unlikely candidate for a primary Aurora A binding site.

To obtain direct evidence of the role of the SARAH domain in the formation of the complex between RASSF1A and Aurora A, we have carried out SPR (Surface Plasmon Resonance) experiments to test the binding of the separately expressed SARAH domain to the immobilised Aurora A kinase domain (Fig. 5a). Since all protein expression and purification were only successful with an N-terminal MBP-tag, we have also carried out control binding experiments with MBP alone. While, as expected, only very weak (possibly aspecific) interaction of MBP with the Aurora A kinase domain could be detected, leading to a negligible binding signal (not shown), the SARAH domain was found to be a specific binding partner of the Aurora A kinase domain. Unfortunately, our binding experiments were partially disturbed by irreversible aggregation on the binding surface, resulting in inability to reach the binding equilibria and determine the *K*_d_ values. Similar effects were also observed with the other two investigated RASSF1A forms, i.e. for ΔN and RBD (Fig. 5b). The definitive observation is that both the formation and the dissociation of the complex with Aurora A are significantly slower for ΔN than for RBD (Fig. 5b), possibly due to the additional interactions formed by ΔN through its SARAH domain. Naturally, the isolated SARAH domain exhibits the smallest binding signal (Fig. 5a) compared to ΔN and RBD (Fig. 5b).

### Structural modelling of the Aurora A – RASSF1A complex

To assist with the interpretation of the kinetic and binding results above, we have carried out modelling studies to determine a possible mode of molecular interaction between Aurora A and its substrate RASSF1A. Since our experiments were restricted to the truncated variants of RASSF1A, i.e. ∆N, ∆N-∆loop and RBD, our modelling was also carried out with these variants. Models were built for the RBD-Aurora and ∆N-Aurora complexes based on predicted structures for the RASSF1A variants and experimental structures of Aurora A.

**Figure 6:**
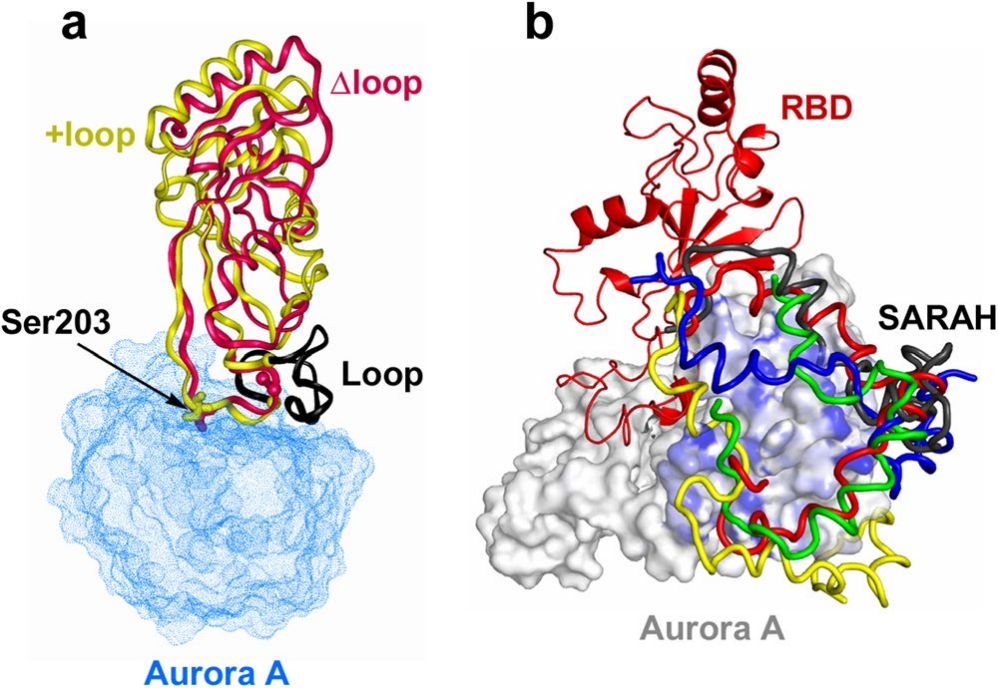
Structural modelling of the complex of Aurora A kinase domain with the RBD (**a**) and ΔN (**b**) fragments of RASSF1A. The kinase domain of Aurora is illustrated by a blue dotted (**a**) and white solid (**b**) surface-covered model. In (**a**) the modelled RBD domain of RASSF1A, as well as its loop-deleted variant are superimposed according to their β-sheets and are shown by yellow and red ribbons, respectively. The two Val residues sequentially bordering the loop are shown as balls, while the loop itself is coloured black. The phosphorylation sites (Ser203) in both variants are represented by stick models. In (**b**), ΔN is shown as a cartoon diagram and only a single chain of RBD (coloured red) is shown, while 5 possible binding configurations of the SARAH domain are coloured red, green, yellow, blue and black, respectively. Blue shading on the surface of Aurora A indicates the probable binding surfaces on the C-terminal lobe as derived from 10 different configurations generated by conformational sampling.

Figure 6a illustrates that the unstructured loop (coloured black) that sequentially precedes the consensus phosphorylation motif (RRTSF) does not interact at all with Aurora A kinase, i.e. the loop itself possibly does not contribute to the formation of the E*S complex. This picture is fully consistent with the kinetic results, i.e. the identical *K*_m_ values obtained for the loop-deletion mutant (∆N-∆loop) and ∆N (Fig. 3 and Table 2). On the other hand, the observed decrease of the *k*_cat_ value upon deletion of the loop could be explained by the loss of flexibility provided by the loop to the nearby phosphorylation site.

Next, the predicted structure of the complex of Aurora A and ∆N (consisting of RBD and the C-terminal SARAH domain) is shown in Fig. 6b. Here, the modelling was performed in agreement with our binding studies (Fig. 5) that the SARAH domain binds to the Aurora A kinase domain, and the goal of the modelling was to explore the possible binding sites. Although we were not able to definitively identify a particular binding site, the modelling results indicate that the binding is possible, and the binding region is the shaded area of the C-terminal lobe of the Aurora A kinase domain as indicated in Fig. 6b.This looks to be a very unique type of interaction that could possibly be formed only with the monomeric form of ∆N.

It is possible that ∆N in itself exists mainly in a dimeric form, as demonstrated by our experiments of size-exclusion chromatography (Fig. 2), which is stabilized through self-interactions between the long helical SARAH domains. Indeed, it has been demonstrated experimentally that SARAH can easily form self-associated dimers, which consist of two well-ordered helices^24^. This is also confirmed by our size-exclusion chromatographic experiments with the isolated SARAH domain (Fig. 2c,d). Upon dissociation of the dimer, however, the helix becomes less ordered, and breaks up into 2 or 3 shorter helices, as suggested by modelling^23^ and experimental^25^ studies. Our experiments and modelling suggest that this less-structured SARAH-helix of the monomeric form of RASSF1A can bind to the kinase domain of Aurora A, as shown in Fig. 6b. The conformation of the SARAH domain may be stabilized upon binding. Whether there is a well-defined binding site for the SARAH domain on the surface of Aurora A or the complex remains “fuzzy”^18,26^ is a question for future investigations.

## Discussion

This work presents the first detailed enzyme kinetic analysis of phosphorylation of the tumour suppressor RASSF1A by human Aurora A kinase. It was found that the relevant fragment (ΔN, after deleting 120 residues from the mainly disordered N-terminal part, cf. Fig. 1) is phosphorylated by Aurora A kinase domain with *k*_cat_ and a *K*_m_ values summarised in Table 2. These values appear plausible as the *k*_cat_ values are about the same magnitude as those previously obtained with Aurora A on various synthetic peptide substrates^12,14,15,27–29^. As for the *K*_m_ values, the data are scarce in the literature, but the value obtained with the peptide substrate Kemptide was around 100–300 μM, indicating a considerably weaker enzyme-substrate interaction compared to the fragment ΔN.

These data and our experiments show that RASSF1A is a more specific substrate for Aurora A compared to the synthetic peptides, as expected. It is known that the phosphorylation site/s (Thr202/Ser203) is/are located next to a disordered loop on the RBD domain of RASSF1A (cf. Figs 1 and 6a). The X-ray structure of the RBD-domain of the homologue RASSF5 (NORE1A) protein has been determined at 1.8 Å resolution, in which the disordered loop could not be resolved^21^. The authors of this paper initially assumed, but finally ruled out, that this loop is required for the binding to Ras. Therefore, instead, the authors further assumed that in the case of the homologue RASSF1A, the loop might be involved in the binding to the kinase Aurora A. We have tested this reasonable assumption by studying the kinetics of phosphorylation by Aurora A in a loop-deleted mutant of the ΔN fragment of RASSF1A (the ΔN-Δloop construct) (Fig. 3a,b). We found a large decrease of the *k*_cat_ value without any change in *K*_m_ (Table 2). These results suggest that the flexible loop of RBD domain of RASSF1A has an important role in the phosphorylation step catalysed by Aurora A. Possibly, it assures the proper conformation of the phosphorylation site, optimal for the phospho-transfer from the γ-phosphate of ATP bound to Aurora A. On the other hand, the loop itself possibly does not contribute to the formation of the E*S complex, since *K*_m_ is not affected by its deletion. These conclusions are further supported by our experiment with a synthetic peptide possessing an amino acid sequence identical to the deleted loop. We have not found any inhibitory effect by this peptide on the phosphorylation reaction (Fig. 3c). Our structural modelling studies on the complex of Aurora A kinase domain and the ΔN part of RASSF1A (Fig. 6a), in fact, illustrate the absence of interaction between the loop and the kinase domain, which is fully consistent with these findings.

A further remarkable finding of this work is the existence of a relatively fast dimer-monomer equilibrium in the case of the investigated N-terminal truncated mutant (ΔN) of RASSF1A (cf. Fig. 2b). This is surprising because an earlier study suggested that the N-terminal part of RASSF1A is required for dimerization^30^. Our gel-chromatographic experiments (Fig. 2a,b) clearly indicate relatively fast dimerization equilibrium only for the ΔN construct but not for RBD. Thus, dimerization of ΔN most probably occurs through the interaction of the SARAH domain which is absent in RBD. Our size-exclusion chromatographic experiments with the isolated SARAH domain confirm this suggestion (Fig. 2c,d). In fact, there are several examples of dimerization of various proteins/enzymes possessing SARAH domains, including RASSF1A^22,23,25,31–36^. The dimerization equilibrium, detected in the present work, however, seems to be much slower compared to the relatively short time-scale of the kinetic measurements. Thus, both the monomeric and the dimeric states of ΔN could be separately characterised kinetically (Fig. 4). Unfortunately, no data on the physiological concentration of RASSF1A is available in the literature, but our data indicate weaker E*S interaction with a significantly higher *K*_m_ value in the case of the dimeric form of RASSF1A ΔN (Table 2). This could be explained by the lack of an available SARAH domain in the dimer. It seems reasonable, therefore, that the SARAH domain is responsible for the specific interaction with the kinase domain of Aurora A.

The interaction between the SARAH domain of RASSF1A and the Aurora A kinase represents a novel interaction type as known interactions mediated by SARAH domains form by SARAH-SARAH association^24,25^, unlike in our case where a SARAH domain is found to bind to a globular domain. Our structural modelling illustrates a possible way of this interaction (Fig. 6b). This model suggests that the SARAH domain tilts towards Aurora A, binds to it in a kinked conformation, and locks it in place, effectively stabilizing the E*S complex. In fact, kinking of the SARAH helix has been also suggested by a previous modelling study^23^. The exact mode of binding, including the question whether Aurora A has a well-defined binding site for the SARAH domain or the binding is more “fuzzy”^18,26^ remains to be investigated.

## Methods

### Materials

The vector used for the expression of proteins was an altered version of the commercially available pET24c vector. The isotope-labelled γ-^32^P-ATP was purchased from Izotóp Intézet Kft. (Hungary). The unlabelled ATP was Sigma-Aldrich product. A synthetic peptide with the sequence corresponding to the deleted loop (cf. below) was synthesized by GenicBio Ltd. Company. All other chemicals used were commercially available, high purity products.

### Mutagenesis of RASSF1A

The following truncated variants of RASSF1A were used in the experiments: ∆N (residues 121–340), RBD (residues 121–290) and ∆N-∆loop (lacking the phosphorylation loop, cf. below). The SARAH domain (residues 291–340) was also expressed separately. Aurora A was also produced as a truncated construct, consisting of only its kinase domain (residues 107–403). The genes encoding the truncated protein mutants were created by using Polymerase Chain Reaction (PCR) to amplificate the appropriate regions of their respective wild-type genes. The primers contained restriction cleavage sites, so the PCR products could be cloned into a modified pET24c vector with a Maltose Binding Protein (MBP)-coding sequence upstream, and 6XHis tag coding sequence downstream to the multiple cloning sites. The RASSF1A loop deletion (∆N-Δloop) was introduced into the ∆N construct by whole plasmid PCR. The primers were designed to stick to sequences bordering the deletion region from both sides but lacking the target sequence (positions coding for residues 177–197, i.e. the sequence PSSKKPPSLQDARRGPGRGTS). After the reaction finished, the mixture was treated by DpnI to get rid of the methylated parental plasmids, leaving the PCR products – carrying the deletion mutants – intact. The sequences of all mutants were confirmed by sequencing.

### Protein expression and purification

All proteins were expressed with N-terminal MBP, and C-terminal 6XHis affinity tags, in *E*. *coli* Rosetta 2 cells using a modified pET24c vector. Cell cultures were grown on 37 °C, to an OD^600^ of 0.6–0.8 then cooled to 21 °C. Expression was induced by addition of 0.4 mM Isopropyl β-D-1-ThioGalactopyranoside (IPTG), and then continued overnight at 21 °C. Cells were harvested by centrifugation, resuspended in a buffer containing CompleteUltra protease inhibitor mix and lysed by ultrasound. The lysate was centrifuged, and the target protein was purified from the supernatant.

All proteins were fused to an N-terminal MBP-tag, and were first purified using amylose affinity chromatography. The bound target proteins were eluted by 10 mM maltose. In the case of the Aurora A kinase domain, this step was followed by a nickel affinity chromatography (by the C-terminal 6XHis tag), to separate the intact protein from its degradation products. This time 250 mM imidazole was used as eluent. All RASSF1A protein constructs have been identified by using SDS PAGE according to their molecular masses of 69, 63, 67 and 51 kDa for ΔN, RBD, ΔN-Δloop and the SARAH domain, respectively (including the MBP-tag).

All proteins were further purified using size exclusion chromatography on a Superose 6 column to separate the native product from the aggregate. The column buffer was 25 mM HEPES, 300 mM NaCl, 3 mM DTT (except for the RBD, which lacks cysteine), at a pH of 7.4. The purified proteins were concentrated, and their final concentrations determined by UV spectrophotometry using molar absorbances calculated on the basis of a previously published method^37^. The solutions were aliquoted, frozen in liquid nitrogen and stored at −80 °C.

Cleavage of the N-terminal MBP-tag by Tobacco Etch Virus (TEV)-protease was also tried in a single case of a truncated variant of RASSF1A followed by a cation exchange chromatography, however, the protein yield was dramatically diminished.

### Analytical gel filtration of the investigated RASSF1A constructs

Samples of different RASSF1A mutants were diluted in a buffer (25 mM HEPES, pH 7.4, 300 mM NaCl) to different concentrations. Of these dilutions, volumes of 100 μl were injected to a column filled with 30 ml Superose 6 gel filtration medium, and chromatographed using FPLC. Proteins were detected by measuring the absorbance of the eluate at 280 nm.

### Kinetic assay of ^32^P-incorporation into the RASSF1A constructs by Aurora A

To examine the initial velocities of phosphorylation of the different RASSF1A variants by Aurora A, reaction mixtures were prepared in a pH 7.4 HEPES buffer, with Aurora A kept at a constant concentration of 40 nM and the concentration of RASSF1A varied. The mixtures also contained 100 mM NaCl, 5 mM MgCl_2_ and 2 mM DTT. The reaction was started by addition of 0.4 mM ATP, partly labelled with ^32^P isotope on its γ-phosphate. To determine the initial velocity of phosphorylation, reaction mixtures were incubated for 2 minutes at 25 °C, and then a sample was pipetted into a reducing SDS sample buffer, terminating the reaction. The samples were boiled, and then excess ATP was separated from the phosphorylated RASSF1A by SDS PAGE.

^32^P incorporation was visualized by exposing the gels to a GE Healthcare StoragePhosphor screen, then scanning the screen by a Typhoon TRIO+ scanner. The resulting image showed bands corresponding to the phosphorylated proteins, with their density related to the amount of phosphate incorporated during enzyme reaction. The densities were quantified using densitometry, and then converted to molar concentration using an appropriate calibration standard of known amounts of fully-phosphorylated RASSF1A. From these concentrations *v*_0_ values for each reaction could be determined – the samples were taken at the initial phase of the reactions, confirmed by the amount of substrate converted.

Using the Michaelis-Menten model of enzyme kinetics, *v*_0_ and [S]_0_ values were plotted and fit using the software Sigma-plot (version 11.0) to determine the kinetic parameters of the enzyme reaction for each variant of RASSF1A.

### Measurement of binding between RASSF1A constructs and the Aurora A kinase domain

SPR binding experiments were carried out to test the binding of the isolated SARAH domain and the ΔN and RBD variants to the immobilised kinase domain of Aurora A using a Biacore X equipment. Aurora A kinase domain was immobilised as a ligand to a GE Healthcare CM5 chip. The different RASSF1A variants were used as analytes in the mobile phase. The signal was automatically corrected by that of a reference cell without any immobilised proteins. All experiments were carried out in a HBS-EP buffer (10 mM HEPES pH 7.4, 150 mM NaCl, 3 mM EDTA and 0.005% Tween-20) at a temperature of 25 °C.

### Structural modelling of the molecular interactions of Aurora A with the RASSF1A mutants

#### RASSF1A model building

A homology model for the ΔN RASSF1A was constructed using the I-TASSER pipeline^38^. Chain B of PDB entry 3ddc^21^, a structure of murine RASSF5, was found to be the best template with a sequence identity of 0.58 over the aligned part, with a coverage of 0.6. The estimated TM-score of the best model was 0.43. Five models were built; these mainly differed in the conformation of the 177–197 loop region. Upon visual inspection, the structure with the most plausible loop conformation was chosen for further modeling.

#### Constructing a model for the RASSF1A-Aurora A complex

The building of a complex of ΔN RASSF1A with the Aurora A kinase required modeling the interaction of the substrate binding site of Aurora A with the phosphorylated residue of RASSF1A. Because there is no Aurora A structure with a bound peptide substrate, we used the structure of cAMP-dependent protein kinase in complex with PSP20, a 20-residue phosphorylated peptide (PDB entry 4ib0^39^) to model the kinase-peptide complex. The Aurora A structure 4dee^40^ (containing an ADP molecule) was fitted onto chain A of 4ib0 using TM-align^41^ and the PSP20 peptide was copied over into the Aurora A structure. Torsion angles of a 14-residue segment of PSP20 (7–20) were used as dihedral restraints for the homologous 193–206 segment of RASSF1A (containing the phosphorylated site), and short (50 ps) vacuum molecular dynamics simulation was carried out with these restraints on our ΔN RASSF1A model. GROMACS 5.0.2^42^ was used with the CHARMM27 forcefield with default parameters at 300 K. The purpose of this simulation was to force the 193–206 segment of ΔN RASSF1A to a structure identical to that of the 7–20 segment of PSP20 in 4ib0. After this occurred, the ΔN RASSF1A-Aurora A complex model was constructed by a least-squares superposition of the ΔN RASSF1A 193–206 segment to the 7–14 segment of the PSP20 peptide that was previously copied into the Aurora A structure. This initial complex structure was used for further simulations.

#### Modeling the possible binding of the RASSF1A SARAH domain to Aurora A

The complex generated in the previous step was constructed without a SARAH domain. To add a SARAH domain, we used Modeller^43^ with a RASSF5 SARAH structure from an earlier modeling study^23^ as a template (KS3 in Fig. 4c in that paper; coordinates obtained as a courtesy of Ruth Nussinov). This structure occurred most frequently in the earlier study, and has a kink in the α-helix. To generate a large number of conformations with different SARAH domain conformations and orientations, geometric simulations were used. The FRODAN program^44^ was used to generate 10,000 different conformations which represent a broad sampling of the conformational space. The 10 structures with the largest interface areas with the Aurora A subunit were used for further modeling. To add an ATP molecule to the models, the ADP molecule in the 1mq4 Aurora A structure^40^ was replaced by the ATP taken from PDB entry 4wb5^45^ (a protein kinase A structure) after superposing the kinase structures, and the Aurora A subunit in all 10 model complexes was replaced by this 1mq4-ATP complex. The 10 Aurora A-ΔN RASSF1A complexes were then subjected to 50 ns molecular dynamics simulations at 300 K in vacuum using GROMACS 2016^42^. The Gromos54a7 forcefield^46^ was used; electrostatic interactions were treated using the Particle Mesh Ewald method^47^ and the LINCS algorithm was used to constrain all bonds^48^. The last structure from each trajectory was then subjected to energy minimization.

## Data Availability

The data that supports the findings of this study are available on request from the corresponding authors, Mária Vas and István Hajdú.
